# The Personal Suicide Stigma Questionnaire (PSSQ): Relation to Self-Esteem, Well-Being, and Help-Seeking

**DOI:** 10.3390/ijerph20053816

**Published:** 2023-02-21

**Authors:** Brant R. Maclean, Tahni Forrester, Jacinta Hawgood, John O’Gorman, Jurgita Rimkeviciene

**Affiliations:** 1 Australian Institute for Suicide Research and Prevention, WHO Collaborating Centre for Research and Training in Suicide Prevention, Griffith University, Brisbane, QLD 4122, Australia; 2 Suicide Research Centre, Institute of Psychology, Faculty of Philosophy, Vilnius University, LT-03100 Vilnius, Lithuania

**Keywords:** personal stigma, self-esteem, PSSQ reliability, suicidality, help-seeking

## Abstract

Two studies are reported that extend the evidence base for use of the Personal Stigma of Suicide Questionnaire (PSSQ). In the first study (N = 117), the Rosenberg Self-Esteem Scale, the WHO-5 measure of well-being, as well as measures of suicidality were examined in relation to the PSSQ. A self-selected sub-sample (N = 30) completed the PSSQ after an interval of two months. In line with the stigma internalization model, when demographic variables and suicidality were accounted for, the PSSQ self-blame subscale was the most significant predictor of self-esteem. As for well-being, the rejection subscale was involved as well as self-blame. The retest stability of the PSSQ for the sub-sample was 0.85 and coefficient alpha for the total sample was 0.95, indicating both good stability and internal consistency for the scale. In the second study (N = 140), PSSQ was studied in relation to intention to seek help from four sources in the case of suicidal ideation. The strongest relationship with PSSQ was with intention not to seek help from anyone (r = 0.35). When other variables were included in the prediction of help-seeking from a general medical practitioner, family or friends, or from nobody, the only significant PSSQ correlate was minimization. For help-seeking from a psychologist or psychiatrist, the most significant predictor was judged helpfulness of prior contact with them. The results from these studies strengthen previous findings of the construct validity of the PSSQ and point to its utility in understanding barriers to help-seeking among those experiencing suicidality.

## 1. Introduction

Stigma originally meant a bodily mark indicative of disease, but over time has come to mean a mark of shame or discredit that, as Goffman [1] observed, can be associated with a group identity, such as that conferred by imprisonment, mental illness, or suicidal behaviour. Stigma, in its current sense, can control social interaction, leading to diminished quality of life and impairment of social functioning for those stigmatized [2]. The stigma of mental illness has been the most widely studied within a framework proposed by Corrigan [3] that distinguishes public stigma from self-stigma [4]. Public stigma involves stereotyping, prejudice, and discrimination directed to a group, whereas self-stigma involves members of the stigmatized group applying those public beliefs to themselves and identifying with them. Internalizing stigma in this way was reported by Livingston and Boyd [5] to relate negatively to outcomes such as hope, self-esteem, and empowerment and, importantly, to adherence to treatment. 

Less research has been directed to the stigma of suicidal behaviour, although it can affect well-being and can act as a barrier to necessary care [6]. Sheehan et al. [7] argued that those surviving a suicide attempt bear a double effect: both the stigma associated with mental illness and that associated with suicidal behaviour. Although some individuals with mental illness report suicidal ideation or attempt suicide, suicidal behaviour is not itself a mental illness [8], and the stigma associated with it needs to be studied separately from the stigma of mental illness. A recent systematic review by Nicholas and colleagues [9] on the measures of suicide stigma indicated a recent increase in interest in suicide stigma measures. However, they report that the majority of the scales in this area are focused on the public level of stigma; more understanding is needed of the personal stigma of suicide, which includes direct experiences of stigma from those with a lived experience of suicidality. Furthermore, they highlighted the need for more studies into construct and discriminant validity of the scales, as opposed to study of only their factor structure.

One of the few scales that assesses suicide stigma from the perspective of the individual experiencing it is the Personal Suicide Stigma Questionnaire (PSSQ) [10]. The term personal stigma refers to: (a) perceived stigma, the individual’s beliefs about public attitudes to suicidality; (b) experienced stigma, the individual’s experiences of actual discrimination and prejudice; and (c) self-stigma, the person’s own internalization of public stigma [4]. One of the strengths of the PSSQ is the inclusion of a lived experience perspective in its design, which is frequently lacking in the development of suicide stigma scales [9]. Rimkeviciene et al. [11] began with the results of a qualitative study of the understanding of stigma by patients attending a clinic specialized in suicide-specific care and by those treating them. They used the themes that emerged to write items for a self-report questionnaire [10]. Item analysis yielded a 16-item scale, with high internal consistency (Cronbach alpha = 0.95) that showed a three-component structure using Principal Component Analysis. The first two subscales (rejection and minimization) are a mixture of perceived and experienced stigma, including items relating to being rejected and to having suicidal thoughts and behaviour invalidated by others. The self-blame subscale captures self-stigma, with items attributing responsibility to oneself. Therefore, the scale includes not only assessment of attitudes, but also the experiences of stigmatizing behaviours, frequently omitted in other scales [9]. The three-factor structure of the scale was confirmed using Confirmatory Factor Analysis in a larger community sample (N = 3947) and showed scalar invariance for age and gender [12]. 

Because the PSSQ is relatively new, there is still limited data on its construct and discriminant validity. Scores on the PSSQ have been found to relate positively to an index of suicidality (r = 0.68) and to a measure of mental illness stigma (r = 0.30) [10]. The correlation with suicidality was high, as expected, but the correlation with mental illness stigma was not so high as to call into question the separateness of the construct of personal suicide stigma. In a second study, PSSQ remained a significant predictor of distress after suicidality and demographic variables were accounted for [12], indicating the specificity of the effects of stigma, as measured by the PSSQ. However, to date, no studies have examined the relation of the PSSQ to positive aspects of functioning, such as well-being and self-esteem, or to its possible role in help-seeking behaviour.

In seeking to extend the construct validity of the PSSQ, we drew on the model proposed by Corrigan and colleagues [13,14] to account for the way mental illness stigma becomes internalized and impairs functioning of the individual. They proposed that individuals first experience negative attitudes and discrimination in society, which lead to their awareness of the generalized attitude toward people like them, or “stereotype awareness.” There follows a three-stage process of the internalization of the harmful stereotype: agreement with it, alignment of self with it, and reduction of self-esteem as a result. Once stigma is internalized through this process, it leads to further adverse outcomes for the individual [5].

A similar process of internalization of stigma, we argue, applies to suicide stigma. Becoming aware of the social stigma of suicidal behaviour can lead the person to agreement and concurrence with the stereotype, and this in turn can lead to reduction in self-esteem and subsequently to other impacts on well-being and the likelihood of seeking help for the behaviour. We further posit that the minimization and rejection subscales of the PSSQ encompass perceptions and experiences of stigma and reflect awareness of the suicide stereotype, and that the self-blame subscale assesses self-stigma, which, by Corrigan and colleagues’ [13] definition, includes agreement and concurrence with the stereotype. If this reasoning is correct, we would expect that self-blame, which reflects the process proximal to self-esteem decrement, will correlate more strongly with self-esteem than the other two subscales assessed with the PSSQ, and that this will also be true for the more generalized effects of self-esteem reduction, such as well-being and help-seeking. 

We examined this formulation of personal stigma of suicide in two studies. Study 1 examined the links between the PSSQ and its subscales, on the one hand, and self-esteem and well-being, on the other. In a second study, the links between stigma and help-seeking were examined. A second study was necessary because of the need to control for other factors that can potentially influence help-seeking.

Because no previous studies had examined the retest reliability of PSSQ, a second aim of Study 1 was to address this gap by re-administering the test two months after the first administration. Stability of scores over time is of particular importance where a test is being used to assess change as a consequence of an intervention. A test that yields unstable scores cannot accurately reflect reliable change. We chose two months as an interval that was unlikely to allow simple recall of previous responses and of the duration of counselling or psychoeducational interventions (6–10 weeks) that might be employed to reduce stigma. Our expectation was that the retest reliability of the PSSQ is at least “good” according to standards proposed by Cicchetti [15]; that is, in the range 0.60 to 0.74. 

## 2. Study 1

### 2.1. Method

#### 2.1.1. Participants

Only participants who reported previous suicidal ideation or behaviour were included in this study, because experience of suicidality is considered necessary for the formation of personal stigma of suicide. In estimating sample size, we expected at least a medium effect size (r = 0.3) for correlations, given the findings of Livingston and Boyd [5] related to mental illness stigma. Because four bivariate correlations (PSSQ with each of two suicidality measures and with each of the well-being and self-esteem measures) were to be tested for statistical significance, to protect the error rate, the Bonferroni adjustment was applied. This meant that with the nominal value of alpha at 0.05, each test was set to be made at *p* < 0.0125 (0.05/4). According to GPower [16], with power of 0.8 and alpha at 0.0125, to detect a correlation of 0.3, a sample size of 99 is required. We allowed for a 20% attrition rate (16% in the previous study using the same method of sampling by Rimkeviciene et al. [10]), and thus set sample size at 119. In fact, this study was terminated when N = 161, but the actual attrition rate (27%) was higher than estimated. A total of 25 participants commenced the survey but did not complete any of the demographic items or any of the psychological questionnaires, and 19 completed only the demographic items. Of the remaining 117, there were some missing data for 16 participants.

Approximately two months after the initial administration (mean = 61.6 days, median = 60 days, SD = 2.45, range = 59 to 70 days) participants were asked to complete the PSSQ again. A total of 30 participants made themselves available.

#### 2.1.2. Measures

The first section of the survey battery included demographic items relating to age, gender (scored 1 for female and 0 for male), and education (scored as a 5-level variable from 1 “incomplete high school” to 5 “post-graduate study”). Relationship status was reduced to a two-level variable, single (coded 0) and partnered (coded 1), which was also the case for employment, employed (coded 1) and unemployed (coded 0). In addition, three questions to gauge potential impacts of the COVID-19 pandemic were included but their analysis is not included in this report.

The PSSQ [10] consists of 16 items divided into three subscales: minimization, rejection, and self-blame. Each item has a 5-point Likert scale, from 0 (*Never*) to 4 (*Very Often*); total scores thus range from 0 to 64, with higher scores reflecting greater stigma.

The Suicidal Behaviour Questionnaire (SBQ-R) was used to assess overall suicidality. It consists of four items that assess life-long history of ideation and attempts, frequency of suicidal ideation, threats of suicide, and likelihood of suicide completion [17]. The total score on the measure ranges from 3 to 18, with higher scores reflecting greater risk for suicidal behaviours. A score of 7 is the cut-off for non-suicidal samples and 8 for clinical samples [17]. The SBQ-R has good internal consistency (Cronbach α = 0.80 to 0.87).

The Suicidal Ideation Attributes Scale (SIDAS) [18] is a 5-item scale that was used to assess current suicidal ideation. Total score ranges from 0 to 50, with a higher score equating to higher severity of suicidal ideation, and a score higher than 21 indicating that the respondent is at high risk of engaging in future suicidal behaviour [18].

The Rosenberg Self-Esteem Scale (RSES) [19] is the most widely used self-esteem measure in the literature and has been a prominent measure of self-esteem in suicidal populations [20,21]. It is a 10-item scale that has demonstrated good test–retest reliability and internal consistency, with Cronbach alpha coefficients regularly reported above 0.8 [22].

The WHO-5 [23] is a five-item self-report measure of subjective well-being that is among the most widely used well-being measures. It has shown good internal consistency (0.85) [24]. Scores range from 0 to 100, with lower scores indicating poorer subjective well-being than higher scores [25].

#### 2.1.3. Procedure

Participants for this study were recruited via multimedia advertising, using social media and an email distribution system, which included a number of Australian suicide prevention stakeholders. In addition, to maximize participation, participants were given the opportunity to enter a draw to win one of three $50 gift vouchers. The advertisement for this study indicated the rationale for the research, eligibility criteria, and a link for further information on this study (e.g., informed consent, anonymity, benefits and risks, prize draw guidelines and rules, and a link to enter the survey). The survey was set up in Research Electronic Data Capture (REDCap), which is a secure online portal for distribution of surveys for research purposes [26].

Item 1 of the SBQ-R was used to screen for past suicidality experiences. The survey was expected to take 25–30 min to complete. Due to the sensitive nature of suicide, and the associated content included in the survey, help and support services were provided throughout the duration of the survey. Participants could withdraw at any time.

This study was approved before commencement by the University Human Ethics Committee (GU Ethics Ref: 2020/386).

#### 2.1.4. Data Analysis

Those completing the whole survey were compared with those who completed only the demographic section and who skipped the psychological questionnaires in terms of the demographic variables to test for differences between the two samples.

For participants who had completed more than the demographic items, only 1.9% of values were missing. Because Little’s [27] Missing Completely at Random Test was not statistically significant, suggesting that the missingness was completely at random and not missing at random, and because the amount of missing data was less than 5% [28], listwise deletion rather than multiple imputation was used to deal with the missing cases.

Pearson correlation was used to examine bivariate relations among the PSSQ and the measures of suicidality, self-esteem, and well-being. Lee and Preacher’s [29] program was used to test the difference between dependent correlations. Hierarchical regression analysis was used to examine the multivariate relations. With well-being or self-esteem as the criterion, control variables (age, gender, education, relationship status, and employment status) were entered at the first step, followed by suicidality measures, SBQ-R and SIDAS at the second step, and PSSQ subscales at the third step. As noted above, the six critical null hypothesis tests were made at alpha = 0.0125. Other tests were made at a nominal conventional alpha = 0.05, but given that a number of tests were made, particularly in the case of the regression analyses, the actual error rate would be greater than that. Regression diagnostics (including checks on normality of error distributions, multicollinearity, heteroscedasticity, independence of errors) were run to examine the satisfactoriness of the models. To examine retest reliability, an ICC was calculated using a two-way mixed effects model with a definition of consistency.

### 2.2. Results

Of the 117 participants, one participant identified as non-binary, with three identifying as “other gender identity,” and one preferring not to say. Data on the gender variable were omitted for these 5 participants. Individual ages (in years) ranged from 18 to 71; 72 (61.5%) of the participants were employed and 58 (49.6%) were partnered.

Those who did not complete the psychological test section of the survey did not differ from those who did in terms of age [t(103) = 0.94, *p* = 0.35], gender [χ^2^(1, N = 105) = 0.07, *p* = 0.79], education [t(103) = 1.32, *p* = 0.19], relationship status [χ^2^(1, N = 105) = 0.05, *p* = 0.82], or employment [χ^2^(1, N = 105) = 0.01, *p* = 0.92]. Those who provided a second test result did not differ from those who did not in terms of age [t(115) = 0.63, *p* = 0.53], gender [χ^2^(1, N = 115) = 0.01, *p* = 0.92], education [t(115) = 0.43, *p* = 0.67], relationship status [χ^2^(1, N = 115) = 0.23, *p* = 0.63], or employment [χ^2^(1, N = 115) = 1.23, *p* = 0.27].

Table 1 presents means, standard deviations, and product moment intercorrelations for all variables in this study and the Cronbach alpha coefficients for each of the psychological tests.

Inspection of Table 1 indicates that there were medium to large positive correlations, significantly different from zero (*p* < 0.001), between PSSQ and the two suicidality measures and large and negative correlations between PSSQ and well-being and self-esteem. Inspection also shows that, as predicted, the self-blame subscale showed the strongest correlation with self-esteem, followed by rejection. The difference between these two coefficients was statistically significant (z = 2.9165, *p* = 0.0035). Self-blame also showed a stronger correlation with well-being than that between minimization and well-being, but in that case the difference was not statistically significant (z = 1.404, *p* = 0.1602).

Table 2 and Table 3 summarize the regression analyses performed with well-being and self-esteem scores as the criteria.

Inspection of the tables shows that PSSQ subscales made a substantial and statistically significant contribution to variance in self-esteem and well-being. Specifically, self-blame, but not minimization or rejection, remained significant predictors of self-esteem when demographic and suicidality variables were accounted for. In addition, suicidality measures did not show significant relationships with self-esteem once personal suicide stigma was accounted for. As for well-being, both self-blame and rejection remained statistically significant predictors of well-being once suicidality and demographic variables were accounted for. Current suicidality remained a significant predictor of well-being after inclusion of the PSSQ subscales.

The single-measures ICC, examining consistency over the 2-month period, was 0.86, 95% CIs [0.735, 0.933]. For the subscales of the PSSQ the values were: for rejection 0.86 [0.728 to 0.931]; for minimization 0.74 [0.524, 0.861]; and for self-blame 0.69 [0.443, 0.839].

## 3. Study 2

An ongoing problem in suicide prevention is putting those in need of help in touch with professional services [30]. In their review of possible barriers to help-seeking, Hom et al. [30] identified stigma as playing a role directly, as well as more subtly, in reducing individuals’ use of professional suicide prevention services. Calear et al. [31] in a large sample survey reported that suicide stigma and suicide literacy (knowledge of symptoms, causes, and interventions for suicidal ideation and behaviour) were associated with help-seeking attitudes and intentions. They used a short form of the Stigma of Suicide Scale (SOSS) [32] to assess public stigma and a short form of the Literacy of Suicide Scale (LOSS) [33] to assess suicide literacy. An adaptation of the General Help-Seeking Questionnaire (GHSQ) [34] was used to assess help-seeking intentions with respect to suicide. Scores on both the SOSS and LOSS were associated at a statistically significant level with aspects of intention to seek help and attitudes toward help-seeking. In general, attitudes were more positive and intentions stronger with greater literacy and lower stigma. The authors noted that their study examined public stigma and that self-stigma may have an important role to play in help-seeking.

The present cross-sectional study examined both suicide literacy, using the SOSS, and suicide stigma, using the PSSQ, in relation to help-seeking as assessed with the GHSQ, as adapted by Calear et al. [31]. We expected to replicate the essential findings of Calear et al. [31] and thus show the practical value of the PSSQ in understanding one of the barriers to help-seeking. More specifically, as outlined earlier, we expected higher scores on the PSSQ and in particular the subscale of self-blame, representing agreement and self-concurrence with the stereotype, to relate to lower intentions to seek help from professional sources (psychologist/psychiatrist and general practitioner) and from family and friends, and greater intention to seek help from nobody.

We also included an assessment of whether or not participants had previously sought professional help and how useful that had been for them. This was based on the four-item prior counselling measure (PCM) described by Wilson et al. [34]. In their study of high school students, rated quality of previous professional contact correlated with intentions to seek help for suicidal thoughts. Wilson et al. [35] found, in a university sample of participants, that 51% had previously sought help from a mental health professional for personal problems. Previous help-seeking was positively correlated with likelihood of seeking help subsequently and, for those who had sought help previously, the judged helpfulness of the experience correlated positively with the likelihood of seeking help again. We expected previous positive experience with professional help to predict help-seeking.

### 3.1. Method

#### 3.1.1. Participants

As in Study 1, participants were screened for past suicidal ideation or behaviour. We used the same logic as in Study 1 and G*Power 3 [16] to estimate a required sample size of 99 (r = 0.3, alpha = 0.0125, one-tailed, power = 0.8) but allowed for a higher attrition rate (30%) in view of the experience of Study 1. Data collection was stopped at 187 participants.

#### 3.1.2. Measures

This study included the 16-item PSSQ for assessment of personal suicide stigma as described in Study 1. In addition to age and gender, participants were asked about their educational background, which was reduced to a two-level variable: having had education/experience in the mental health field (coded 1) and no education/experience in the mental health field (coded 0).

The GHSQ, as adapted by Calear et al. [31], was used to assess help-seeking intentions if they were to experience suicidal thoughts. Five sources of help were listed (psychologist/psychiatrist, general Practitioner, family or friend, nobody, and the Internet) but only the first four of these were used in the analysis of results, consistent with the approach of Calear et al. Participants were asked to indicate on a 4-point scale how likely/unlikely they were to seek help from each of the sources.

The PCM [34,35] comprises four items that relate to whether the participant has ever seen a mental health professional (counsellor, psychologist, psychiatrist) for assistance with a personal problem; how many visits they have made; the type of health professional they saw; and how helpful they perceived this assistance. The latter was rated on a 5-point scale from “extremely helpful” to “extremely unhelpful.” Only responses to the first and fourth questions were used in the present study.

The 27-item version of the LOSS [36] was used, but based on more recent data on scale validity [37], item “Suicide rarely happens without warning” was removed. Each of the items on the LOSS is responded to on a 3-point scale (true, false, or I don’t know). Correct responses are allocated a score of 1 and incorrect or “I don’t know” responses assigned a score of 0 and then summed. Total scale scores range from 0 to 26, with higher scores indicative of higher suicide literacy. Cronbach alpha for the scale in the present sample was 0.82.

#### 3.1.3. Procedure

This study used the same online software platform (REDCap) and participant recruitment procedure as in Study 1. In a departure from Study 1, based on ethical concerns, the participants were advised through the survey that completion of all questions was voluntary to ensure that forcing an answer to a particular question was not distressing for the participant. Upon completion of the survey, participants were provided with an information page of options for support if the participant was affected by the survey items. Participants were advised that the survey included 59 questions and would take approximately 25 min to complete. The final survey page included a link for participants who chose to enter the prize draw (one of three $50 gift vouchers). This took them to a new page not linked to the survey where they could provide contact details.

Prior to conducting this study, it was approved by the University Research Ethics Committee (GU Ethics Ref: 2021/443).

#### 3.1.4. Missing Values and Data Analysis

Possibly because of the permissive instructions, coupled with the opportunity to enter the prize draw, there was a substantial amount of missing data. Of the 187 cases registered on the data base, 35 provided no responses to any of the questions, 9 provided responses only to the demographic questions, and the data for 3 were deleted because they indicated on the screening question that they had not experienced suicidal thoughts. Of the remaining 140, missing values ranged from 2.1% to 9.3% for the help-seeking items, from 12.9% to 15.7% for the previous experience questions, from 13.6% to 15.7% for the PSSQ items, and from 22.1% to 24.3% for items on the LOSS. Listwise deletion would have resulted in the loss of 61 participants.

Little’s MCAR test was statistically significant [χ^2^ (df = 1334) = 1424.957, *p* = 0.041], indicating that the data were more likely missing at random than missing completely at random. Multiple imputation [38,39] was implemented using the procedure in SPSS Version 28, with 10 imputations followed by analysis using procedures that allowed pooling of the output. Variables included for imputation comprised all demographics and item responses for each of the GHSQ, PCM, LOSS, and PSSQ. Total scores on questionnaires were computed after imputation. Comparison of variables in terms of mean, variance, skewness, and kurtosis for the original dataset and the pooled results indicated little difference. The analyses reported here are for the pooled results following imputation.

Pearson correlation was used to examine bivariate relations among the help-seeking intentions and other measures. Hierarchical multiple regression analyses were performed using each of the four sources of help as an outcome. The demographic factors were entered at the first step, followed by LOSS score and PSSQ subscales at the final step. For those who reported prior treatment from mental health professionals, additional regression analyses were run predicting seeking help from psychologist/psychiatrist and nobody, adding rated helpfulness of previous professional contact at the second step in addition to LOSS. When the multiple imputation option is employed, SPSS 23.0 does not provide tests for the statistical significance of increments in the variance accounted for at each step or beta coefficients for comparisons of variables, so only tests of the statistical significance of unstandardized regression coefficients are reported. Regression diagnostics (including checks on normality of error distributions, multicollinearity, heteroscedasticity, independence of errors) were run to examine the satisfactoriness of the models.

### 3.2. Results

Of the sample of 140 participants, 118 (84.3%) reported they had seen a professional mental health worker for a personal problem and offered an assessment of the helpfulness of the experience. Table 4 summarizes the means, standard deviations, and intercorrelation of outcome measures of intention to seek help from each of the four sources and the demographic and predictor variables. The subscale scores of the PSSQ are also included.

Inspection of the table indicates that rated helpfulness of previous experience was the strongest predictor of intention to seek help from a psychologist/psychiatrist, and that PSSQ score was not significantly correlated with this source of help. For the other three sources of help, PSSQ and one or two of its subscales were significant predictors. For intention to seek help from nobody, PSSQ was a stronger predictor than rated helpfulness of previous professional contact. The LOSS scale was not significantly correlated with any of the sources of help.

Results of the regression models are summarized in Table 5 and Table 6.

Inspection of Table 5 indicates that PSSQ was a statistically significant predictor for three of the four outcomes at the final step in the regression analysis; that is, when all the other predictors were controlled. Significant predictors for seeking help from psychologists/psychiatrists were mental health education or experience, in that those with this education/experience were less likely to seek help. The only significant PSSQ scale was minimization, with those with higher experiences of minimization less likely to seek help from a general practitioner or family/friends and more likely to seek help from nobody. Other significant predictors for seeking help from family/friends were age and gender: younger individuals and males were less likely to seek help. For those with prior experience with mental health professionals (Table 6), judged helpfulness of prior contact and PSSQ rejection subscale were significant predictors of seeking help from a psychiatrist/psychologist, in that those with more positive prior experiences and higher levels of rejection experiences were more likely to seek help. When all other variables were accounted for, PSSQ minimization was the only significant predictor for seeking help from nobody, with those with higher minimization scores more likely to seek help from nobody.

## 4. Discussion

The PSSQ was developed to assess personal stigma associated with suicidal ideation and behaviour. Previous studies demonstrated that the PSSQ has good psychometric properties, including a clear and reproducible factor structure and good internal consistency, and that it correlates with measures of reported suicidal ideation and suicide attempt [10,12]. These present studies replicated previous findings that the PSSQ has good internal consistency and a clear relation to suicidal ideation and behaviour, and extended these findings in demonstrating that:

- Scores on the PSSQ and its subscales show stability over a two-month period;
- The PSSQ relates at a non-trivial level to measures of self-esteem and well-being, both independently and when the contribution of suicidality and demographic variables are taken into account;
- The self-blame subscale of the PSSQ is the major correlate in the case of self-esteem, but in the case of well-being both self-blame and rejection are involved;
- The PSSQ predicts help-seeking, in the case of suicidal ideation, from a general practitioner, family and friends, or from no one, but not help-seeking from a psychologist or psychiatrist, in which case previous positive experience is the statistically significant predictor;
- The minimization subscale of the PSSQ, and not the self-blame subscale, is the stronger predictor of help-seeking.

These new findings speak to the reliability of the PSSQ and its subscales and to the utility and construct validity of the scale.

The data on retest reliability over a two-month period indicated a high degree of agreement between PSSQ scores on the two occasions. The lower bound of the 95% confidence interval places the estimate for this sample within the range Cicchetti [15] described as good and the upper bound within the excellent range. This indicates that the PSSQ is not only internally consistent but is stable, at least up to two months, and may be appropriately used when studying change in score as a result of an intervention, such as psychoeducation directed to reducing personal stigma. The values were lower and the confidence intervals were wider for the subscales, indicating caution in the use of these.

In exploring construct validity of the PSSQ, we adapted the model proposed by Corrigan and colleagues [13,14] for stigma in the case of mental illness to personal suicide stigma. We hypothesized that the PSSQ self-blame subscale reflects a process of agreement and concurrence with the stereotype of the suicidal person and that this is responsible for reduced self-esteem. Consistent with this hypothesis, the self-blame scale showed the strongest correlation of the subscales with self-esteem, and unlike the other two subscales remained the only statistically significant predictor of self-esteem when demographic and suicidality variables were accounted for. However, the relationship between PSSQ and well-being was more complex. As predicted, self-blame was the strongest correlate of well-being, although the correlation with well-being was not statistically different from that for rejection and well-being. Self-blame remained a significant predictor of well-being when the other variables were accounted for, but rejection had a statistically significant beta weight as well, and a positive rather negative sign as in the zero-order correlation. It is important to note that this relation emerged only when all the other factors, including self-blame, were partialed from the relation. One potential hypothesis for such a finding is that those participants who are more aware of rejection from others due to stigmatization but do not attribute it to themselves (instead, as Corrigan et al. [13] describe it, forcefully reacting to the injustice), may have better well-being outcomes compared to those who do not recognize rejection or internalize it. However, until the effect is replicated, this unexpected result can also be attributed to an artefact of regression with a number of variables in the equation.

The differences with respect to the two outcome variables might result from the breadth of the constructs involved, with well-being a more conceptually complex outcome. In this regard, it is noteworthy that current suicidal ideation remained a significant predictor of well-being once demographic variables and stigma were accounted for. This indicates that the PSSQ is not redundant with indices of suicidality but, more importantly, indicates that well-being is not a simple function of stigma but reflects the severity of suicidality. This in turn suggests that targeting only personal stigma without help with the experiences of suicidality is unlikely to be a sufficient intervention.

We expected PSSQ score to predict intention to seek help beyond the prediction provided by LOSS and the judged helpfulness of previous experience of help from professional sources, which had been shown to be significant in previous studies [31,34,35]. We further expected that, in line with the stigma internalization model [13], self-stigma in the form of self-blame would be the most significant predictor of help-seeking. The results only partially confirmed the first part of the hypothesis and disconfirmed the second part. PSSQ score was not significantly related to help-seeking from a psychologist or psychiatrist, but related inversely at a statistically significant level to help-seeking from a general practitioner and from family or friends, and positively and at a statistically significant level with intention to seek help from nobody. The strongest correlation for the PSSQ was with help-seeking from nobody, suggesting an isolating effect of the personal stigma captured by the PSSQ. Minimization (perceptions and experiences that others minimize the importance of suicidality), and not self-blame as predicted, was the only PSSQ subscale that remained a significant predictor of intention to seek help when other variables were accounted for. This implies that failure to be taken seriously in the past leads to a negative attitude to seeking help in the future, best expressed in the generalized response of intending to seek help from no one. This is in line with findings from a qualitative study that found that one of the fears keeping young adults from seeking help from GPs is that one’s struggles might be minimized by the healthcare provider [40]. Similarly, assessment of the effects of a stigma-reduction intervention showed that self-rated decreases of stigma were not always related to more openness to seek help [41]. From the viewpoint of the stigma internalization model [13], a possible hypothesis is that experiences of suicide stigma in terms of minimization of suicidality are sufficient to reduce help-seeking behaviour, irrespective of whether this attitude is internalized or not. However, a further study with a longitudinal design assessing the stigma internalization process would be needed to confirm this.

Help-seeking from a psychologist or psychiatrist seems to be less likely if a person has mental health education or work experience. However, positive prior contact with a mental health worker was the strongest predictor of intention to seek help in the future, irrespective of mental health literacy or self-stigma. This suggests a hypothesis that an increase in the uptake of mental health services for people with suicidality is better achieved by organizing opportunities to have a positive contact with mental health specialists, such as brief contact interventions [42], rather than public suicide literacy or de-stigmatization campaigns.

In general, the PSSQ and its subscales have been shown to have some important correlates, but the mechanisms underlying these relations are not clear. The adaptation of the Corrigan model had some success but the network of relations needs further explication.

### Limitations

These studies have certain limitations. They used a cross-sectional design and thus are uninformative with respect to the direction of effects. For example, it is unclear whether personal stigma results in lower self-esteem or whether lower self-esteem gives rise to personal stigma. Both effects may of course be occurring, with self-esteem affecting personal stigma which in turn influences self-esteem. Such reciprocal interaction is to be expected in personality functioning but can only be addressed with difficulty and only in longitudinal designs [43].

A further limitation is that the psychological variables employed here all employed self-report as the method of measurement. Common method variance is known to inflate correlations among variables [44] and this may account for some of the shared variance in the present study. It is difficult, however, in the case of suicidal ideation to suggest alternatives to self-report, and such is the case as well for self-esteem and well-being, because these are all subjective, private experiences. We simply note that the “true” correlation for the underlying constructs, operationalized here by self-report variables, may be lower than that found here.

The samples were relatively small and self-selected. Although sample sizes were in line with pre-study power calculations, participants volunteered for this study, which may well have produced bias. Participants who volunteer, particularly for a study on suicidality, may differ from those who do not and thus are not representative of the wider population. More importantly, not all those who initially volunteered actually completed this study. The attrition rate was higher than expected, which we attributed to the use of an attractive incentive and the failure to provide a mechanism for ensuring that participants completed all sections of the survey before being eligible for the prize draw. Some may well have avoided completing the psychological test items because of the time involved or because of the content, but by at least commencing the survey they became eligible. We compared those who completed with those who did not based on the information that we had available, and no differences were detected, but it remains possible that there were differences that we were not able to test. Whether the results generalize to other populations, such as patients presenting to hospital emergency departments following a suicide attempt or patients seeking treatment for suicidal behaviour awaits further examination. A larger sample and longer intervals between testing would provide more data on test–retest reliability.

## 5. Conclusions

The network of relations demonstrated in these and previous studies, limitations notwithstanding, point to the construct validity of the PSSQ and its subscales as a measure of the personal stigma associated with suicidal thinking and behaviour. As such, they provide support for use of the PSSQ to gauge the impact of suicide stigma when working with people experiencing suicidality. Assessment of a person’s position on the scale or subscales can be a point of departure for exploration of issues relevant to the person’s experience of stigma and self-worth. The scale may also be useful in tracking changes in personal stigma over the course of counselling or psychoeducation.

## Figures and Tables

**Table 1:** Means, standard deviations, and intercorrelations between PSSQ Score, the measures of suicidality, self-esteem, well-being, and the demographic variables (N = 107).

Items	1	2	3	4	5	6	7	8	9	10	11	12	13
1. PSSQ	(0.95)												
2. Rejection	0.86 *	(0.92)											
3. Minimization	0.83 *	0.70 *	(0.90)										
4. Self-Blame	0.83 **	0.66 **	0.57 **	(0.93)									
5. SBQ-R	0.61 **	0.57 **	0.50 **	0.50 **	(0.78)								
6. SIDAS	0.55 **	0.45 **	0.47 **	0.50 **	0.60 **	(0.85)							
7. RSES	−0.71 **	−0.54 **	−0.51 **	−0.71 **	−0.53 **	−0.49 **	(0.90)						
8. WHO-5	−0.50 **	−0.34 **	−0.40 **	−0.51 **	−0.41 **	−0.60 **	0.62 **	(0.90)					
9. Age	−0.03	0.14	−0.03	−0.03	0.14	−0.04	0.17	−0.08					
10. Gender	0.32 **	0.39 **	0.25 **	0.20 *	0.30 **	0.11	−0.20 *	−0.24 *	0.11				
11. Education	−0.19 *	−0.15	−0.16	0.23 *	−0.07	−0.19 **	0.23 *	0.21 *	0.12	0.06			
12. Relationship	−0.07	−0.02	0.03	−0.16	0.09	−0.16	0.02	0.08	0.02	0.12	0.28 **		
13. Employment	−0.29 **	−0.26 **	−0.26 **	−0.24 *	−0.24 **	−0.28 **	0.28 **	0.29 **	0.00	−0.02	0.24 **	0.15	
*M*	49.42	13.08	13.53	22.79	13.67	12.89	23.74	9.38	37.31	0.77	3.50	0.50	0.62
*SD*	15.72	5.51	4.58	7.82	4.78	11.89	6.13	5.57	12.91	0.42	1.23	0.50	0.49

Note: 2–4 refer to subscales of PSSQ; coefficients in brackets are Cronbach alphas. * Correlations statistically significant at the level *p* < 0.05; ** Correlations statistically significant at the level *p* < 0.01.

**Table 2:** Hierarchical regression analysis with self-esteem as the criterion (n = 100).

	Model 1			Model 2			Model 3		
	B	SE	β	B	SE	β	B	SE	β
Constant	17.82	2.8		25.75	2.76		35.68	3.05	
Age	0.09	0.05	0.181	0.09 *	0.04	0.194	0.06	0.04	0.134
Gender	−3.11 *	1.34	−0.215	−1.27	1.2	−0.087	−0.53	1.08	−0.036
Education	1.03	0.52	0.196	0.65	0.46	0.124	0.37	0.39	0.071
Relationship	−0.3	1.18	−0.024	−0.02	1.04	−0.002	−1.04	0.9	−0.084
Employed	2.97 *	1.24	0.236	1.24	1.1	0.107	0.79	0.95	0.062
SBQ-R				−0.47 ***	0.14	−0.360	−0.23	0.12	−0.179
SIDAS				−0.11 *	0.06	−0.21	−0.03	0.05	−0.059
PSSQ Rejection							−0.04	0.13	−0.036
PSSQ Minimization							−0.05	0.14	−0.033
PSSQ Self-Blame							−0.42 ***	0.08	−0.515
*R* ^2^		0.201 **			0.421 ***			0.598 ***	
Δ*R*^2^		0.201 **			0.22 ***			0.177 ***	

* *p* < 0.05, ** *p* < 0.01, *** *p* < 0.001.

**Table 3:** Hierarchical regression analysis with well-being as the criterion (n = 101).

	Model 1			Model 2			Model 3		
	B	SE	β	B	SE	β	B	SE	β
Constant	7.31	2.35		12.28	2.38		17.51	2.99	
Age	−0.02	0.04	−0.041	−0.02	0.04	−0.058	−0.06	0.04	−0.134
Gender	−2.95 *	1.17	−0.238	−2.08	1.07	−0.167	−2.33 *	1.1	−0.188
Education	0.92 *	0.46	0.204	0.55	0.41	0.121	0.5	0.4	0.11
Relationship	0.1	1.05	0.01	−0.3	0.95	−0.027	−0.63	0.94	−0.057
Employed	2.55 *	1.1	0.229	1.35	1	0.121	0.95	0.99	0.085
SBQ-R				0.02	0.12	0.014	0.06	0.13	0.048
SIDAS				−0.23 ***	0.05	−0.493	−0.2 ***	0.05	−0.436
PSSQ Rejection							0.28 *	0.14	0.289
PSSQ Minimization							−0.21	0.15	−0.169
PSSQ Self-Blame							−0.21 *	0.08	−0.291
*R* ^2^		0.182 **			0.384 ***			0.439 ***	
Δ*R*^2^		0.182 **			0.202 ***			0.056 *	

* *p* < 0.05, ** *p* < 0.01, *** *p* < 0.001.

**Table 4:** Means and standard deviations of predictors and correlations with the four sources of help in Study 2.

			Correlations with Likelihood of Seeking Help from:			
Predictor	Mean	SD	Psychologist/ Psychiatrist	GP	Family/Friend	Nobody
Age	44.27	15.02	0.064	0.073	−0.248 **	0.051
Gender ^1^	1.77	0.42	−0.014	−0.053	−0.147	0.114
MH education or experience ^2^	0.21	0.41	−0.130	−0.061	−0.067	0.145
LOSS	18.68	4.67	0.169	0.096	−0.005	0.116
Judged helpfulness of previous professional help	3.47	1.11	0.337 **	0.093	0.045	−0.202 *
PSSQ Rejection	13.86	5.79	0.014	−0.170	−0.270 **	0.281 **
PSSQ Minimization	12.57	4.48	−0.058	−0.333 **	−0.323 **	0.374 **
PSSQ Self-Blame	22.46	7.81	−0.108	−0.145	−0.225 *	0.281 **
PSSQ Total	49.07	15.93	−0.065	−0.231 **	−0.305 **	0.351 **

^1^ Scored 1 for female and 0 for male. ^2^ Scored 1 for present, 0 for absent. * Correlations statistically significant at the level *p* < 0.05; ** Correlations statistically significant at the level *p* < 0.01.

**Table 5:** Summary of hierarchical regression analyses for each source of help as the outcome variable.

	Psychiatrist/Psychologist				General Practitioner				Family/Friends				Nobody			
Variable	B	SE	*t*	*p*	B	SE	*t*	*p*	B	SE	*t*	*p*	B	SE	*t*	*p*
(Constant)	2.16	0.63	3.42	0.001	2.86	0.60	4.77	0.000	5.03	0.57	8.83	0.000	0.49	0.60	0.83	0.410
Age	0.00	0.01	0.33	0.745	0.00	0.01	0.58	0.561	−0.02	0.01	−3.80	**0.000**	0.00	0.01	0.65	0.515
Gender	0.01	0.25	0.04	0.969	−0.04	0.24	−0.17	0.868	−0.50	0.22	−2.32	**0.020**	0.17	0.23	0.75	0.451
MH education or experience	−0.47	0.24	−1.98	**0.048**	−0.24	0.23	−1.04	0.297	−0.07	0.22	−0.34	0.731	0.33	0.23	1.45	0.148
LOSS	0.04	0.02	1.80	0.074	0.02	0.02	1.24	0.216	0.00	0.02	−0.06	0.954	0.03	0.02	1.31	0.194
Rejection	0.04	0.03	1.28	0.200	0.02	0.03	0.72	0.472	0.02	0.03	0.71	0.478	−0.01	0.03	−0.52	0.603
Minimization	−0.03	0.03	−0.81	0.417	−0.10	0.03	−3.20	**0.001**	−0.07	0.03	−2.24	**0.026**	0.06	0.03	2.16	**0.031**
Self-Blame	−0.02	0.02	−1.19	0.233	0.00	0.02	0.16	0.872	−0.02	0.02	−1.38	0.169	0.03	0.02	1.62	0.107

Note: MH = mental health; Rejection, minimization, and self-blame refer to the PSSQ subscales; *p* levels significant at 0.05 level marked in bold.

**Table 6:** Summary of hierarchical regression analyses for those who had prior contact with mental health professionals for each source of help as the outcome variable.

	Psychiatrist/Psychologist				Nobody			
Variable	B	SE	*t*	*p*	B	SE	*t*	*p*
(Constant)	0.95	0.65	1.46	0.144	1.04	0.63	1.66	0.098
Age	0.00	0.01	−0.29	0.770	0.01	0.01	0.96	0.338
Gender	−0.06	0.23	−0.24	0.814	0.20	0.23	0.89	0.374
MH education or experience	−0.41	0.22	−1.85	0.064	0.30	0.23	1.31	0.191
LOSS	0.02	0.02	1.05	0.295	0.03	0.02	1.64	0.106
Judged helpfulness of prior contact	0.40	0.09	4.28	**0.000**	−0.18	0.10	−1.92	0.057
PSSQ Rejection	0.06	0.03	2.33	**0.020**	−0.03	0.03	−0.97	0.334
PSSQ Minimization	−0.03	0.03	−1.04	0.299	0.07	0.03	2.27	**0.023**
PSSQ Self-Blame	−0.02	0.02	−1.17	0.244	0.03	0.02	1.56	0.120

Note: MH = mental health; *p* levels significant at 0.05 level marked in bold.

## Data Availability

Because of the sensitive nature of the questions related to suicidality and stigma, permission to make full dataset available online was not sought from the participants and the full dataset is not made available online due to ethical reasons. It can, however, be accessed upon a reasonable request from the last author.
